# Can Alterations in Cerebrovascular CO_2_ Reactivity Be Identified Using Transfer Function Analysis without the Requirement for Carbon Dioxide Inhalation?

**DOI:** 10.3390/jcm12062441

**Published:** 2023-03-22

**Authors:** Shigehiko Ogoh, Hironori Watanabe, Shotaro Saito, James P. Fisher, Erika Iwamoto

**Affiliations:** 1 Department of Biomedical Engineering, Toyo University, Kawagoe 350-8585, Japan; 2 Neurovascular Research Laboratory, University of South Wales, Pontypridd CF37 1DL, UK; 3 Department of Physiology, Faculty of Medical and Health Sciences, University of Auckland, Auckland 1023, New Zealand; 4 School of Health Sciences, Sapporo Medical University, Sapporo 060-8556, Japan

**Keywords:** CO_2_ inhalation, time response, magnitude response, transfer function analysis, brain blood flow

## Abstract

The present study aimed to examine the validity of a novel method to assess cerebrovascular carbon dioxide (CO_2_) reactivity (CVR) that does not require a CO_2_ inhalation challenge, e.g., for use in patients with respiratory disease or the elderly, etc. In twenty-one healthy participants, CVR responses to orthostatic stress (50° head-up tilt, HUT) were assessed using two methods: (1) the traditional CO_2_ inhalation method, and (2) transfer function analysis (TFA) between middle cerebral artery blood velocity (MCA V) and predicted arterial partial pressure of CO_2_ (PaCO_2_) during spontaneous respiration. During HUT, MCA V steady-state (i.e., magnitude) and MCA V onset (i.e., time constant) responses to CO_2_ inhalation were decreased (*p* < 0.001) and increased (*p* = 0.001), respectively, indicative of attenuated CVR. In contrast, TFA gain in the very low-frequency range (VLF, 0.005–0.024 Hz) was unchanged, while the TFA phase in the VLF approached zero during HUT (−0.38 ± 0.59 vs. 0.31 ± 0.78 radians, supine vs. HUT; *p* = 0.003), indicative of a shorter time (i.e., improved) response of CVR. These findings indicate that CVR metrics determined by TFA without a CO_2_ inhalation do not track HUT-evoked reductions in CVR identified using CO_2_ inhalation, suggesting that enhanced cerebral blood flow response to a change in CO_2_ using CO_2_ inhalation is necessary to assess CVR adequately.

## 1. Introduction

An increase in the partial pressure of arterial carbon dioxide (PaCO_2_) leads to a dilatation of the cerebral blood vessels. This mechanism of cerebral blood flow (CBF) regulation is termed cerebrovascular CO_2_ reactivity (CVR) and functions alongside the central respiratory chemoreflex to help maintain brain pH homeostasis [1,2,3]. Increases in PaCO_2_ lower brain pH, which modulates enzyme and ion channel activity [4], meaning that CVR is important for maintaining brain function [1]. Accordingly, the assessment of CVR is used worldwide to directly evaluate brain vascular health [5]. For example, an impaired CVR is related to mild cognitive impairment [6] and Alzheimer’s disease [7], and has been identified in cardiovascular, cerebrovascular, and neurological disorders [8,9,10,11]. These previous findings suggest that CVR measurements have clinical utility in the prediction of brain disease.

Several methodologies have been established to identify CVR in humans using time-domain analysis [2,12,13,14,15,16,17,18] and frequency-domain analyses (e.g., transfer function analysis (TFA), autoregressive moving average analysis, or support vector machines) [3,19,20,21,22,23]. In these methodologies, a CO_2_ inhalation challenge has been used to accurately determine CVR via an increase in the response range of CBF because fluctuations in CO_2_ are relatively small during spontaneous respiration without CO_2_ inhalation. However, CO_2_ inhalation may cause acute stress that produces intense behavioral and physiological responses in humans [24], such as transient elevations of blood pressure (i.e., the fight-or-flight response) [23,25]. Acute stress can have a vasoconstrictive effect on the peripheral vasculature [26], with the potential to indirectly modify CVR. In addition, CO_2_ inhalation can cause anxiety, which has been reported to enhance CVR [27]. CO_2_ inhalation also causes hyperventilation via the respiratory chemoreflex [2], which modifies the CBF response to a change in CO_2_ [28]. Therefore, it is possible that these physiological responses induced by CO_2_ inhalation modify CVR independent from hypercapnia-induced direct cerebral vasodilation. Moreover, CO_2_ inhalation for identifying CVR has both financial and time implications [29]. Given these issues, a method for accurately determining CVR without the need for CO_2_ inhalation would be advantageous, such as for patients with respiratory disease or the elderly, etc. The use of TFA to quantify the relationship between spontaneously occurring fluctuations in CBF and the partial pressure of end-tidal CO_2_ (P_ET_CO_2_), akin to the assessment of dynamic cerebral autoregulation [30], may provide an easy (no intervention) and stress-free method for determining CVR. 

Given this background, the aim of the present study was to examine the validity of the method (TFA) under spontaneous respiration without CO_2_ inhalation for assessing CVR. In our recent study [31], we observed that orthostatic stress (50° head-up tilt, HUT) attenuated CVR determined by traditional methods (i.e., magnitude response or time response, *τ*). In the present study, the change in CVR during 50° HUT from supine was assessed using the TFA between middle cerebral artery blood velocity (MCA V) and predicted PaCO_2_ during spontaneous respiration (at the operating point) to compare with CVR indices determined using the traditional methods with a CO_2_ inhalation challenge. We hypothesized that the CVR determined by TFA without CO_2_ inhalation attenuated during HUT if this new method is valid to determine CVR.

## 2. Materials and Methods

### 2.1. Ethics

The experimental protocol was approved by the Institutional Review Board at Toyo University (Approval Number: 2019-045) and each participant provided written informed consent before participation. The study was undertaken in accordance with the principles of the Declaration of Helsinki.

### 2.2. Participants

Twenty-one healthy adults participated in this study (13 men and 8 women; age, 23 ± 3 years; stature, 166.8 ± 9.6 cm; body mass, 59.4 ± 12.4 kg, mean ± standard deviation). All participants were non-smokers, free of any cerebrovascular and/or cardiovascular diseases, and were not taking any over-the-counter and/or prescribed medications. Before the experiment, participants were required to abstain from caffeinated beverages, strenuous exercise, and alcohol for 24 h [32]. Furthermore, the participants were instructed to consume a light meal at least 4 h before the start of the experiment. 

### 2.3. Study Design

All measurements were performed on the same day for each participant. CVR was determined in two body positions: supine and 50° head-up tilt (HUT). The order of the supine and 50° HUT conditions was randomized for each participant. After instrumentation, participants were placed on the tilt table. It takes a few minutes for fluid shifts to reach equilibrium following a change in body position [33], and positional changes also alter pulmonary ventilation [34], taking ~7–8 min to reach steady-state subsequent to chemoreflex activation [35]. Therefore, each condition began with a 20 min rest, following which 5 min of baseline data were obtained while participants spontaneously breathed room air. After the baseline recording was completed, participants were switched to inspiring a gas mixture from a Douglas bag containing 5% CO_2_ and 21% O_2_, balanced with N_2_. The hypercapnia induced by a rapid change in the F_I_CO_2_ lasted for 12 min to ensure a steady-state equilibration was achieved. After the first condition, the body position was changed (supine to 50° HUT or 50° HUT to supine), and once again participants rested for at least 20 min while inspiring room air. The baseline and hypercapnia protocol were repeated. Room temperature was set at 24–25 °C.

### 2.4. Measurements

Heart rate (HR) was measured using a lead II electrocardiogram (bedside monitor, BMS-3400; Nihon Kohden, Tokyo, Japan). Beat-to-beat arterial blood pressure (ABP) was monitored continuously using a finger photoplethysmography (Finometer Pro; Finapres Medical Systems, Amsterdam, The Netherlands) with a cuff placed on the middle finger of the left hand, which was supported at the level of the right atrium in the mid-axillary line. Mean MCA V (MCA V_mean_), an index of CBF, was measured through the right temporal window using a transcranial Doppler ultrasonography (TCD) system (DWL Doppler Box-X; Compumedics, Singen, Germany). The TCD probe was fixed in position using a dedicated headband (Elastic Headband T; Compumedics) to maintain a constant insonation angle throughout the experiment. For the characterization of respiratory responses to hypercapnia (CO_2_ inhalation), participants breathed through a leak-free face mask attached to a flowmeter and two-way valve. The valve mechanism allowed participants to inspire room air or a gas mixture from a 300 L Douglas bag. Respiratory rate (RR), pulmonary ventilation (V_E_), tidal volume (V_t_), and P_ET_CO_2_ were measured breath-by-breath using an automated gas analyzer (AE-310S, Minato Medical Science, Osaka, Japan). 

### 2.5. Data Analysis

All data were sampled continuously at 1 kHz using an analog-to-digital converter (Power Lab 16 s; AD Instruments, Sydney, Australia) and stored on a laboratory computer for offline analysis. Mean arterial pressure (MAP) and MCA V_mean_ were obtained from each waveform. The predicted PaCO_2_ was derived from P_ET_CO_2_ using the following equation [13]: predicted PaCO_2_ = 2.367 + 0.884 × P_ET_CO_2_. Importantly, a previous study demonstrated that the relationship between P_ET_CO_2_ and PaCO_2_ was unchanged by changes in central blood volume [36]. During supine and 50° HUT, all variables were averaged over 120 s immediately before and at the end of CO_2_ inhalation for steady-state measurements. 

Steady-state change in CO_2_-determined CVR (the magnitude response of CVR): CVR was expressed as the absolute change in MCA V_mean_ per absolute change in PaCO_2_ (cm/s/mmHg) using a linear model: MCA V_mean_ = A + B×predicted PaCO_2_, where the CVR is given by the slope B [12,13]. 

**Figure 1:**
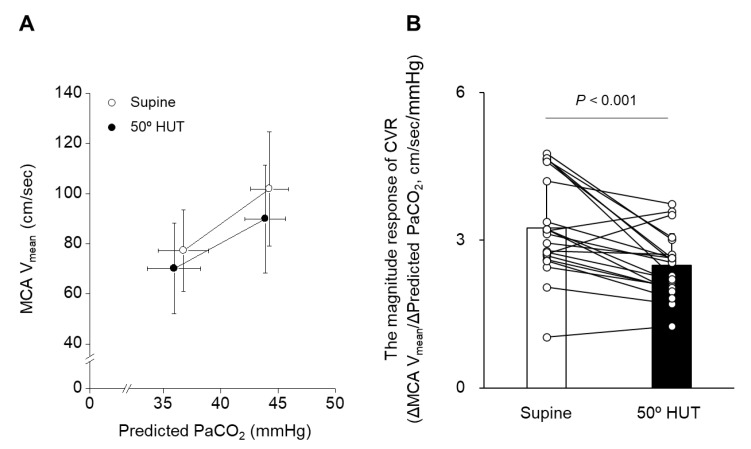
The relationship between middle cerebral artery mean blood velocity (MCA V_mean_) and predicted partial pressure of carbon dioxide (PaCO_2_) (**A**) and the average of the magnitude response of cerebrovascular CO_2_ reactivity (CVR) during supine and 50° head-up tilt (HUT) conditions (**B**) the bar and opened circles represent average and individual values, respectively (*n* = 21; 13 men and 8 women). The *p*-value represents the paired *t*-test analyses between supine and 50° HUT.

Step-change in CO_2_-determined CVR (time constant, the time response of CVR): Dynamic responses of MCA V_mean_ were evaluated using a one-compartment nonlinear least-squares optimization method [16,17,37]. Before analysis, MCA V_mean_ data was resampled at 10 Hz. The onset response of MCA V_mean_ to the CO_2_ inhalation protocol was then fitted to the single-exponential regression equation consisting of the response time latency [CO_2_-response delay (*t*0)], baseline value, gain term (G), and time constant (*τ*): MCA V_mean_ = G × {1 − exp[− (*t* − *t*0)/*τ*]} + MCA V_mean_ 0, where *t* is time, and MCA V_mean_ 0 is a baseline value. Time 0 reflects the start of CO_2_ inhalation (Figure 1A). The “*τ*” is used to determine the time response of CVR, and a prolonged *τ* means a worse/attenuated time response of CVR. 

TFA-determined CVR (TFA gain and phase): TFA analysis between predicted PaCO_2_ and MCA V_mean_ was performed to evaluate the dynamic CBF response to spontaneously occurring changes in CO_2_ during supine and 50° HUT. The 5 min breath-by-breath (predicted PaCO_2_) (mmHg) and beat-to-beat (MCA V_mean_) (cm/s) data without (5 min steady-state data) CO_2_ inhalation were resampled at 4 Hz for spectral analysis [30]. The time series were first detrended with third-order polynomial fitting and then subdivided into 512-point segments with 50% overlap for spectral estimation. For each segment, the linear trend was subtracted and a Hanning window was applied. Following that, frequency-domain transformations were computed with a fast Fourier transformation algorithm. The transfer function H(f) between the two signals was calculated as H(f) = Sxy(f)/Sxx(f), where Sxx(f) is the auto spectrum of changes in predicted PaCO_2_ and Sxy(f) is the cross-spectrum between predicted PaCO_2_ and MCA V_mean_. The transfer function magnitude |H(f)| and phase spectrum |Φ(f)| were obtained from the real part HR(f) and imaginary part HI(f) of the complex function. Moreover, the transfer function H(f) was normalized to the mean values of input (x) and output (y) variables as H′(f) = [Sxy(f)x]/[Sxx(f)y], and the normalized gain was calculated as 20 log H′(f) to provide values in decibels. The TFA coherence, phase, and normalized gain were calculated in the very low (VLF; 0.005–0.024 Hz), low (LF; 0.024–0.15 Hz), and high frequency (HF; 0.15–0.30 Hz) ranges, respectively. This frequency range was determined by previous studies [3,38,39]. Additionally, the dynamic effects of nonlinearities and CO_2_ are more prominent in the VLF range [38,39] and TFA gain between predicted PaCO_2_ and MCA V_mean_ as an index of dynamic CVR gradually decreased with an increase in frequency over a cut-off frequency (0.024 Hz) [3]. Ogoh et al. [3] have for the first time assessed dynamic CVR in all frequency ranges using a binary white noise sequence (0–7% inspired CO_2_ fraction) from the P_ET_CO_2_ to mean MCA V or minute ventilation, respectively. The authors demonstrated that the dynamic characteristics of the relationship between CO_2_ inhalation-induced binary white noise of P_ET_CO_2_ and MCA V were maintained below a cut-off frequency of 0.024 Hz but gradually decreased with an increase in frequency over 0.024 Hz. In the present study, therefore, we focused on the TFA parameters within the VLF range below 0.024 Hz to examine the dynamic CVR during spontaneous respiration.

### 2.6. Statistical Analysis

Data from our pilot study (*n* = 6) were used to perform a prospective power analysis. The critical sample size was estimated to be 19 participants to detect differences in *τ* assessed by MCA V_mean_ between supine and 50° HUT conditions, with an assumed type 1 error of 0.05 and statistical power of 80%. Thus, the sample size of the present study was sufficient to achieve the desired statistical assurance. A linear mixed-effects model with fixed-effects of “Condition (supine vs. 50° HUT)” and/or “Time (with vs. without CO_2_ inhalation)” was used to compare steady-state data. Before the analysis for step-change in CO_2_- and TFA-determined CVR during supine and 50° HUT without (baseline) or with CO_2_ inhalation, the Shapiro-Wilk’s test was applied to verify the normal distribution for each variable. A normal distribution was confirmed in all CVR indices (*W* ≥ 0.911, *p* ≥ 0.057). To compare normally distributed outcomes between supine and 50° HUT, and between without and with CO_2_ inhalation, we incorporated paired samples *t*-tests. The Wilcoxon matched-pairs signed-ranks test was employed where appropriate as a non-parametric equivalent. All data were analyzed using SPSS (IBM SPSS Statistics Version 27.0) and expressed as mean ± standard deviation (SD). Statistical significance was set at *p* < 0.05.

## 3. Results

### 3.1. Hemodynamic Response to Orthostatic Stress (50° HUT)

As expected, 50° HUT caused an increase in HR (*p* < 0.001, Table 1), while MCA V_mean_ (*p* < 0.001), predicted PaCO_2_ (*p* = 0.007), and P_ET_CO_2_ decreased (*p* = 0.009). MAP, RR, V_E_, and Vt were unchanged during HUT (*p* = 0.567, *p* = 0.622, *p* = 0.946, and *p* = 0.900, respectively).

### 3.2. Time Domain Analysis Determined CVR

**Figure 2:**
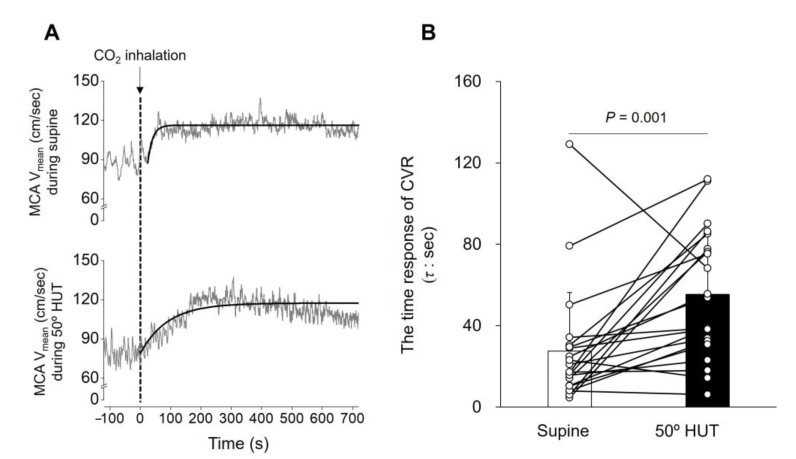
The response of middle cerebral artery mean blood velocity (MCA V_mean_) to 5% CO_2_ inhalation in a representative participant (**A**) and the average of the time response of cerebrovascular CO_2_ reactivity (CVR) during supine and 50° head-up tilt (HUT) conditions (**B**): the bar and opened circles represent average and individual values, respectively (*n* = 21; 13 men and 8 women). The *p*-value represents the Wilcoxon matched-pairs signed-ranks analyses between supine and 50° HUT. *τ,* time constant, as an index of time response of CVR.

The magnitude response of CVR, determined from the MCA V_mean_ response to a steady-state change in CO_2_, decreased from supine to 50° HUT (*p* < 0.001, Figure 1). The time constant (τ) determined from the MCA V_mean_ onset response to step-change in CO_2_ increased from supine (27.5 ± 28.9 s) to 50° HUT (55.4 ± 31.5 s, *p* = 0.001, Table 2 and Figure 2), indicating that the time response of CVR was attenuated by orthostatic stress. Both these results showed that CVR was attenuated during HUT.

### 3.3. TFA-Determined CVR without CO_2_ Inhalation 

**Figure 3:**
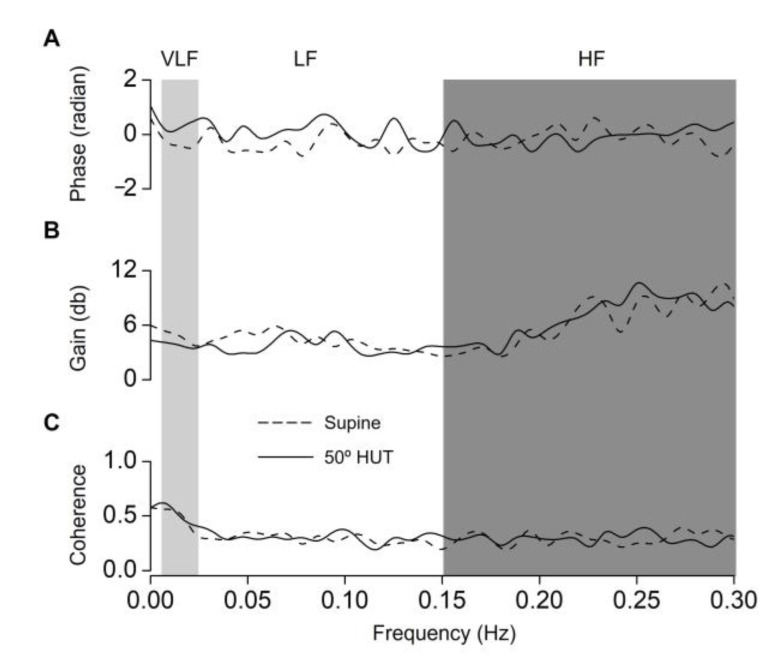
Cross-spectral analysis of the entire spectrum from 0 to 0.30 Hz during supine and 50° head-up tilt (HUT) conditions during spontaneous respiration without CO_2_ inhalation. The group-averaged transfer function phase (**A**), gain (**B**) and coherence function (**C**) between spontaneous changes in predicted partial pressure of arterial carbon dioxide (PaCO_2_) and middle cerebral artery mean blood velocity (MCA V_mean)_ during supine and 50° HUT conditions are shown (*n* = 21; 13 men and 8 women). The dash-dotted and solid lines represent the TFA parameters at supine and 50° HUT, respectively.

TFA phase, gain, and coherence between predicted PaCO_2_ and MCA V_mean_ without CO_2_ inhalation are provided in Figure 3 and Table 3. As shown in Figure 3, coherence was higher than 0.5 in the VLF range, but above 0.024 Hz coherence gradually decreased and was <0.3 in the LF and HF ranges. These findings indicate that TFA in the VLF range rather than other frequency range is useable as an index of CVR. TFA gain in all frequency ranges was unchanged during 50° HUT compared with the supine condition without CO_2_ inhalation, indicating the magnitude of CVR unchanged during HUT, and it was not matched with the magnitude response of CVR determined from the step-change in CO_2_. The VLF TFA phase approached zero during HUT compared with supine (supine: −0.38 ± 0.59 and 50° HUT: 0.31 ± 0.78 radians, *p* = 0.003, Table 3 and Figure 3) during spontaneous respiration without CO_2_ inhalation. This finding of VLF TFA phase indicates that the time response of CVR was increased (not attenuated) during HUT, and it was not matched with the time response of CVR determined from the step-change in CO_2_. Furthermore, LF and HF TFA phases without CO_2_ inhalation were not different between supine and 50° HUT conditions (LF: *p* = 0.064 and HF: *p* = 0.705, Table 3).

## 4. Discussion

In the present study, we assessed whether CVR can be reliably determined using TFA between the predicted PaCO_2_ and MCA V_mean_ without the requirement for CO_2_ inhalation (i.e., during spontaneous respiration), that may be advantageous for patients with respiratory disease or the elderly, etc. As expected, we observed that CVR, determined with the traditional method, using a CO_2_ inhalation challenge was impaired during 50° HUT. However, TFA gain and phase did not represent a similar trend to these traditional CVR indexes during 50° HUT. These findings suggest that TFA (gain and phase) without CO_2_ inhalation under spontaneous respiration does not adequately assess CVR.

As in previous studies [31,39], the magnitude response and time response of CVR (*τ*) determined by CO_2_ inhalation, the traditional index of CVR, were attenuated during 50° HUT (*p* < 0.001, Figure 1 and *p* = 0.001, Figure 2, respectively). The physiological mechanism explaining the orthostatic stress-induced attenuation in CVR remains unknown, but it has been speculated that the attenuated CVR may reflect a progressively reduced cerebrovascular reserve to compensate for the increasingly unstable systemic circulation during orthostatic stress, which could ultimately lead to cerebral hypoperfusion and syncope [31,39]. In addition, orthostatic stress could influence the cerebrovasculature, including ventilation-perfusion matching that is distributed across the zones of the lung during these posture changes and affects PaCO_2_. In the present study, we examined whether TFA-determined CVR was changed during 50° HUT in a similar way to the traditional CVR index to examine the validity of the TFA data without CO_2_ inhalation as an index of CVR. However, TFA gain (all frequencies) without CO_2_ inhalation was not different between the supine and 50° HUT conditions (Table 3). This finding suggests that TFA gain without CO_2_ inhalation does not represent the magnitude response of CVR (i.e., attenuated) as a traditional CVR index. On the other hand, the value of the VLF TFA phase as a dynamic time response was increased but approached zero from supine during 50° HUT to reach a positive value without CO_2_ inhalation (*p* = 0.003, Table 3), indicating that orthostatic stress shortened (i.e., improved) the time response of dynamic CVR. This finding also suggests that the TFA phase did not accurately assess the time response of CVR (*τ*) calculated from the MCA V_mean_ onset response to step-change in CO_2_ (i.e., attenuated) as a traditional CVR index. Collectively, these findings suggest that the TFA phase and gain without CO_2_ inhalation may not be adequate to assess changes in CVR as the traditional index during HUT. 

The main findings of our study are supported by a previous study [40] using blood oxygen level-dependent (BOLD) MRI. This previous study [40] reported that larger CO_2_ stimuli provide a more sensitive means of identifying reductions in the vasodilatory reserve. This suggests that without a sufficiently large stimulus, such as CO_2_ inhalation, the CBF response to CO_2_ may be underestimated. BOLD imaging has the benefit that it gives semi-quantitative information on CVR, and it provides high spatial resolution, which allows the identification of smaller regions of impaired reactivity. However, the BOLD signal itself provides an indirect rather than a direct measure of cerebral perfusion, as provided by newer MRI techniques such as arterial spin labeling. In addition, MRI measurement is expensive and many previous studies used CBF velocity measured by the Doppler method to assess CVR [2,3,12,13,14,16,21,22,23,28]. Importantly, the relative change in BOLD signal is lower than that of CBF velocity determined by the Doppler method [41,42]; therefore, it is possible that under hypercapnic stimulus, the relationship between BOLD signal change and CBF may differ in different individuals [41]. Thus, further investigation may be needed to examine whether small CO_2_ exposures can identify CVR using the Doppler method and TFA technique to minimize the potentially confounding effects of CO_2_ inhalation. 

Previous studies [3,38,43] provided important information regarding the use of frequency domain analysis for assessing CVR. Panerai et al. [43] demonstrated use of a cross-correlation function that spontaneous breath-to-breath changes in P_ET_CO_2_, as well as arterial blood pressure, can contribute significantly to the observed CBF velocity variability. In addition, Mitsis et al. [38] demonstrated use of a multiple input model that arterial pressure fluctuations explain most of the high-frequency blood flow velocity variations (above 0.04 Hz), while P_ET_CO_2_ fluctuations, as well as nonlinear interactions between arterial pressure and CO_2_, have a considerable effect in the lower frequencies (below 0.04 Hz). These findings indicate that the CBF response to a change in CO_2_ is slower than that of a response to a change in arterial pressure. However, the independent contribution of change in P_ET_CO_2_ on cerebrovasculature in the dynamic analysis below 0.04 Hz is unclear because this previous study used a multiple input model. More recently, Ogoh et al. [3] demonstrated the dynamic characteristics of the relationship between CO_2_ inhalation-induced binary white noise of P_ET_CO_2_, and MCA V gradually decreased with an increase in frequency over a cut-off frequency (0.024 Hz). This finding suggests that the reflection of the dynamic relationship between P_ET_CO_2_ and MCA V to TFA data reduces in the range of frequency above 0.024 Hz. Indeed, the coherence was highest in the VLF range and gradually decreased above 0.024 Hz (Figure 3). Thus, the relationship between spontaneous changes in predicted PaCO_2_ and MCA V is statistically stronger in the range of frequency below 0.024 Hz compared with the range from 0.024 to 0.04 Hz. However, in the present study, TFA coherence in the VLF range was close to 0.5, which is an acceptable level of TFA, but slightly below it (supine, 0.47 ± 0.22; HUT 0.50 ± 0.18, Table 2). In addition, the TFA phase and gain in the VLF range without CO_2_ inhalation below 0.024 Hz did not represent a similar change to the CVR determined by the onset of MCA V response to a step-change in CO_2_. These findings suggest that TFA data may not be adequate to assess CVR, especially without CO_2_ inhalation under spontaneous respiration. Importantly, Ogoh et al. [3] used CO_2_ inhalation to determine the dynamic characteristics of the relationship between binary white noise of P_ET_CO_2_ (0–5%) and MCA V. Taken together, CO_2_ inhalation, which enhanced MCA V response to changes in P_ET_CO_2_, appears necessary to assess CVR, even using TFA. 

Potential limitations of the present study warrant careful consideration. First, a change in the diameter of the target vessels (MCA) could modify blood velocity measured using transcranial Doppler independently of flow. MCA diameter is changed during severe CO_2_ inhalation [44,45]. However, the MCA diameter appears to remain relatively constant in humans during moderate variations in blood pressure and CO_2_ [46] and during orthostatic stress [47]. Second, TFA coherence is low, and thus the validity of the TFA method may not be sufficient to identify CVR under spontaneous respiration (at the operating point). Although CO_2_ inhalation did not increase TFA coherence, further studies using techniques to increase TFA coherence (e.g., controlling respiration rate [28]) may be needed to increase the validity of the findings of the present study. Third, the present study examined only one physiological stressor (orthostatic stress) in healthy subjects. Thus, further work is required to verify that the relationships reported between the different methods to assess CVR hold true during other physiological perturbations, or in patients with respiratory disease. In addition, our assessment of TFA was performed using a relatively small sample size, which may increase the probability of a type I error. However, retrospective power calculations indicated that the sample size required to detect a difference in LF TFA gain between supine and HUT conditions would be ~100 participants, tentatively arguing against sample size inflation. Finally, we did not identify the menstrual phases in the female subjects. In female subjects, sex hormones change across the menstrual cycle. However, we assessed the validity of TFA analysis between P_ET_CO_2_ and MCA V under spontaneous respiration as an index of CVR, and the different two methods were applied to each subject in the same menstrual phase. Thus, the changes in sex hormones across the menstrual cycle did not affect the current results. 

### Perspectives and Significance

The traditional methods for determining CVR employ a CO_2_ inhalation challenge to increase the CBF response range to accurately identify CVR, but the changes produced in PaCO_2_ are much larger than the spontaneous changes in PaCO_2_ that occur in everyday life. Along with increasing ventilation via a central chemoreflex [1,3,28], CO_2_ inhalation causes activation of sympathetic nerve activity [48,49]. Moreover, CO_2_ inhalation can cause acute stress [26] and anxiety [27], which affect cerebral vasculature. An important consideration when using CO_2_ inhalation to assess CVR is that it may affect ventilation via a central chemoreflex [1,3,28], and the resultant change in ventilation can modify PaCO_2_, and thus CBF, via the closed-loop feedback system [1,2]. Thus, the time and magnitude responses of CVR may not respond uniformly in all experimental circumstances and populations (e.g., patients and the elderly). For example, the central respiratory chemoreflex, which is a ventilatory response to CO_2_ inhalation, is attenuated in patients with respiratory disease, e.g., chronic obstructive pulmonary disease (COPD) [50,51,52,53], raising the possibility that attenuation of the respiratory chemoreflex overestimates CVR. Thus, some previous studies [54,55,56] demonstrated that CVR attenuated in patients with COPD. However, further investigation is needed to identify how the respiratory response contributes to CVR clinically, especially in patients with respiratory disease. Under these backgrounds, it is possible that CO_2_ inhalation modifies the CBF regulatory system, as well as cerebral vasculature, and, consequently, it may alter the CBF response to change in PaCO_2_ per se. Therefore, determining CVR without CO_2_ inhalation may be better to assess CVR accurately, especially in patients with respiratory disease. Unfortunately, the findings of the present study indicate that the TFA phase and gain cannot be used to assess CVR during spontaneous respiration without CO_2_ inhalation. As such, we may need to establish other methods which do not affect respiratory function, such as spontaneous respiration with low concentration CO_2_ inhalation, to accurately identify CVR.

## 5. Conclusions

The present study, for the first time, identified CVR during spontaneous respiration without CO_2_ inhalation using TFA and assessed whether these TFA data without CO_2_ inhalation are representative of CVR identified using the time domain CO_2_ challenge method (the magnitude and time responses of CVR). These findings indicate that CVR metrics determined by TFA without a CO_2_ inhalation do not track HUT-evoked reductions in CVR identified using CO_2_ inhalation, suggesting that CO_2_ inhalation which enhances CBF response to changes in CO_2_ is necessary to assess CVR adequately. Future research is needed to investigate whether low-concentration CO_2_ inhalation can be used to identify cerebral CO_2_ reactivity in those for whom pronounced hypercapnia is unsuitable.

## Figures and Tables

**Table 1:** Hemodynamic and respiratory variables during spontaneous respiration (free breathing) and CO_2_ inhalation.

	Supine (*n* = 21)		50° HUT (*n* = 21)		Linear Mixed-Effects Model		
Variables					*p*-Values		
	Without CO_2_ Inhalation	With CO_2_ Inhalation	Without CO_2_ Inhalation	With CO_2_ Inhalation	Condition	Time	Interaction
HR, beats/min	64.4 ± 10.7	70.1 ± 12.6	80.5 ± 16.1	83.2 ± 14.7	<0.001	<0.001	0.176
MAP, mmHg	93.0 ± 8.9	97.2 ± 9.8	92.9 ± 9.2	98.6 ± 8.4	0.567	<0.001	0.476
MCA V_mean_, cm/s	77.2 ± 16.3	101.9 ± 22.9	70.1 ± 18.1	89.9 ± 21.5	<0.001	<0.001	0.083
RR, breath/min	16.4 ± 3.7	17.9 ± 5.3	15.9 ± 3.4	17.9 ± 4.8	0.622	0.001	0.690
${\overset{•}{V}}_{E}$, L/min	8.8 ± 1.4	18.6 ± 3.7	8.8 ± 1.4	18.5 ± 4.7	0.946	<0.001	0.985
V_t_, mL	576.4 ± 122.9	1126.4 ± 335.7	583.0 ± 111.7	1110.8 ± 300.6	0.900	<0.001	0.754
P_ET_CO_2_, mmHg	38.9 ± 2.5	47.4 ± 1.9	38.0 ± 2.7	47.0 ± 2.0	0.009	<0.001	0.317
Predicted PaCO_2_, mmHg	36.8 ± 2.2	44.2 ± 1.7	36.0 ± 2.3	43.9 ± 1.8	0.007	<0.001	0.262

All values are means ± SD. HR, heart rate; MAP, mean arterial pressure; MCA V_mean_, mean blood velocity in the middle cerebral artery; RR, respiratory rate; ${\overset{•}{V}}_{E}$, minute ventilation; V_t_, tidal volume; P_ET_CO_2_, end-tidal partial pressure of carbon dioxide; predicted PaCO_2_, predicted partial pressure of arterial carbon dioxide.

**Table 2:** Step-change in CO_2_-determined cerebrovascular CO_2_ reactivity (CVR) at supine and 50° HUT.

Variables	Supine (*n* = 21)	50° HUT (*n* = 21)	*p*-Values
*t*_0_, s	8.5 ± 5.6	10.5 ± 4.6	0.132
*y*_0_, cm/s	77.2 ± 16.3	70.1 ± 18.1	<0.001
G	26.1 ± 10.6	23.0 ± 7.8	0.102
*τ*, s	27.5 ± 28.9	55.4 ± 31.5	0.001

All values are means ± SD. CO_2_ response delay (*t*_0_), response time latency; *y*_0_, baseline value; G, gain term; *τ,* time constant, as an index of time response of CVR. A normal distribution was confirmed for all CVR indices (*W* ≥ 0.933, *p* ≥ 0.160), except for *τ* during supine (*W* = 0.684, *p* < 0.001). *τ* was analyzed using the Wilcoxon matched-pairs signed-ranks test, and the paired samples *t*-test was performed for the other variables.

**Table 3:** Transfer function analysis-determined cerebrovascular CO_2_ reactivity (CVR) during supine and 50° head-up tilt (HUT) without CO_2_ inhalation.

Variables		Supine (*n* = 21)	50° HUT (*n* = 21)	*p*-Values
Phase, radian	VLF	−0.38 ± 0.59	0.31 ± 0.78	0.003
	LF	−0.32 ± 0.61	0.04 ± 0.61	0.064
	HF	−0.12 ± 0.44	−0.07 ± 0.49	0.705
Gain, db	VLF	4.58 ± 1.57	3.75 ± 2.20	0.099
	LF	4.17 ± 1.85	3.69 ± 2.15	0.126
	HF	6.46 ± 4.84	6.90 ± 4.63	0.664
Coherence	VLF	0.47 ± 0.22	0.50 ± 0.18	0.594
	LF	0.37 ± 0.22	0.41 ± 0.17	0.601
	HF	0.31 ± 0.19	0.35 ± 0.10	0.396

All values are means ± SD. VLF, very low frequency; LF, low frequency; HF, high frequency. A normal distribution was confirmed for all CVR indices (*W* ≥ 0.913, *p* ≥ 0.063), except for *τ* during supine, VLF TFA phase during supine, VLF TFA gain during 50° HUT, HF TFA gain during supine and 50° HUT without CO_2_ inhalation (*W* ≥ 0.730, *p* ≤ 0.040). VLF phase, VLF gain, and HF gain were analyzed using the Wilcoxon matched-pairs signed-ranks test, and the paired samples *t*-test was performed for the other variables.

## Data Availability

The data presented in this study are available upon request, due to ethical restrictions.

## References

[1] Ogoh S. (2019). Interaction between the respiratory system and cerebral blood flow regulation. J. Appl. Physiol..

[2] Ogoh S., Hayashi N., Inagaki M., Ainslie P.N., Miyamoto T. (2008). Interaction between the ventilatory and cerebrovascular responses to hypo- and hypercapnia at rest and during exercise. J. Physiol..

[3] Ogoh S., Shibata S., Ito G., Miyamoto T. (2020). Dynamic characteristics of cerebrovascular reactivity or ventilatory response to change in carbon dioxide. Exp. Physiol..

[4] Chesler M. (2003). Regulation and modulation of pH in the brain. Physiol. Rev..

[5] Mandell D.M., Han J.S., Poublanc J., Crawley A.P., Stainsby J.A., Fisher J.A., Mikulis D.J. (2008). Mapping cerebrovascular reactivity using blood oxygen level-dependent MRI in Patients with arterial steno-occlusive disease: Comparison with arterial spin labeling MRI. Stroke.

[6] Richiardi J., Monsch A.U., Haas T., Barkhof F., Van de Ville D., Radu E.W., Kressig R.W., Haller S. (2015). Altered cerebrovascular reactivity velocity in mild cognitive impairment and Alzheimer’s disease. Neurobiol. Aging.

[7] Glodzik L., Randall C., Rusinek H., de Leon M.J. (2013). Cerebrovascular reactivity to carbon dioxide in Alzheimer’s disease. J. Alzheimers. Dis..

[8] Markus H., Cullinane M. (2001). Severely impaired cerebrovascular reactivity predicts stroke and TIA risk in patients with carotid artery stenosis and occlusion. Brain.

[9] Haight T.J., Bryan R.N., Erus G., Davatzikos C., Jacobs D.R., D’Esposito M., Lewis C.E., Launer L.J. (2015). Vascular risk factors, cerebrovascular reactivity, and the default-mode brain network. Neuroimage.

[10] Junejo R.T., Braz I.D., Lucas S.J.E., van Lieshout J.J., Lip G.Y.H., Fisher J.P. (2019). Impaired Cerebrovascular Reactivity in Patients With Atrial Fibrillation. J. Am. Coll. Cardiol..

[11] Smoliński Ł., Członkowska A. (2016). Cerebral vasomotor reactivity in neurodegenerative diseases. Neurol. Neurochir. Pol..

[12] Ainslie P.N., Duffin J. (2009). Integration of cerebrovascular CO_2_ reactivity and chemoreflex control of breathing: Mechanisms of regulation, measurement, and interpretation. Am. J. Physiol. Regul. Integr. Comp. Physiol..

[13] Peebles K., Celi L., McGrattan K., Murrell C., Thomas K., Ainslie P.N. (2007). Human cerebrovascular and ventilatory CO_2_ reactivity to end-tidal, arterial and internal jugular vein PCO_2_. J. Physiol..

[14] Sato K., Sadamoto T., Hirasawa A., Oue A., Subudhi A.W., Miyazawa T., Ogoh S. (2012). Differential blood flow responses to CO(2) in human internal and external carotid and vertebral arteries. J. Physiol..

[15] Tominaga S., Strandgaard S., Uemura K., Ito K., Kutsuzawa T. (1976). Cerebrovascular CO_2_ reactivity in normotensive and hypertensive man. Stroke.

[16] Ogoh S., Ainslie P.N., Miyamoto T. (2009). Onset responses of ventilation and cerebral blood flow to hypercapnia in humans: Rest and exercise. J. Appl. Physiol..

[17] Poulin M.J., Liang P.J., Robbins P.A. (1996). Dynamics of the cerebral blood flow response to step changes in end-tidal PCO_2_ and PO_2_ in humans. J. Appl. Physiol..

[18] Poublanc J., Crawley A.P., Sobczyk O., Montandon G., Sam K., Mandell D.M., Dufort P., Venkatraghavan L., Duffin J., Mikulis D.J. (2015). Measuring cerebrovascular reactivity: The dynamic response to a step hypercapnic stimulus. J. Cereb. Blood Flow Metab..

[19] McKetton L., Sobczyk O., Duffin J., Poublanc J., Sam K., Crawley A.P., Venkatraghavan L., Fisher J.A., Mikulis D.J. (2018). The aging brain and cerebrovascular reactivity. Neuroimage.

[20] Duffin J., Sobczyk O., Crawley A.P., Poublanc J., Mikulis D.J., Fisher J.A. (2015). The dynamics of cerebrovascular reactivity shown with transfer function analysis. Neuroimage.

[21] Chacon M., Araya C., Panerai R.B. (2011). Non-linear multivariate modeling of cerebral hemodynamics with autoregressive Support Vector Machines. Med. Eng. Phys..

[22] Edwards M.R., Devitt D.L., Hughson R.L. (2004). Two-breath CO(2) test detects altered dynamic cerebrovascular autoregulation and CO(2) responsiveness with changes in arterial P(CO(2)). Am. J. Physiol. Regul. Integr. Comp. Physiol..

[23] Regan R.E., Fisher J.A., Duffin J. (2014). Factors affecting the determination of cerebrovascular reactivity. Brain Behav..

[24] Ziemann A.E., Allen J.E., Dahdaleh N.S., Drebot I.I., Coryell M.W., Wunsch A.M., Lynch C.M., Faraci F.M., Howard M.A., Welsh M.J. (2009). The amygdala is a chemosensor that detects carbon dioxide and acidosis to elicit fear behavior. Cell.

[25] Elsaid N., Saied A., Kandil H., Soliman A., Taher F., Hadi M., Giridharan G., Jennings R., Casanova M., Keynton R. (2021). Impact of stress and hypertension on the cerebrovasculature. Front. Biosci..

[26] Karim H.T., Tudorascu D.L., Butters M.A., Walker S., Aizenstein H.J., Andreescu C. (2017). In the grip of worry: Cerebral blood flow changes during worry induction and reappraisal in late-life generalized anxiety disorder. Transl. Psychiatry.

[27] Van den Bergh O., Zaman J., Bresseleers J., Verhamme P., Van Diest I. (2013). Anxiety, pCO_2_ and cerebral blood flow. Int. J. Psychophysiol..

[28] Ogoh S., Suzuki K., Washio T., Tamiya K., Saito S., Bailey T.G., Shibata S., Ito G., Miyamoto T. (2019). Does respiratory drive modify the cerebral vascular response to changes in end-tidal carbon dioxide?. Exp. Physiol..

[29] Duffin J. (2011). Measuring the respiratory chemoreflexes in humans. Respir. Physiol. Neurobiol..

[30] Claassen J.A., Meel-van den Abeelen A.S., Simpson D.M., Panerai R.B., on Behalf of the international Cerebral Autoregulation Research Network (CARNet) (2016). Transfer function analysis of dynamic cerebral autoregulation: A white paper from the International Cerebral Autoregulation Research Network. J. Cereb. Blood Flow Metab..

[31] Watanabe H., Saito S., Washio T., Bailey D.M., Ogoh S. (2022). Acute Gravitational Stress Selectively Impairs Dynamic Cerebrovascular Reactivity in the Anterior Circulation Independent of Changes to the Central Respiratory Chemoreflex. Front. Physiol..

[32] Burma J.S., Macaulay A., Copeland P., Khatra O., Bouliane K.J., Smirl J.D. (2020). Comparison of cerebrovascular reactivity recovery following high-intensity interval training and moderate-intensity continuous training. Physiol. Rep..

[33] Ogoh S., Volianitis S., Nissen P., Wray D.W., Secher N.H., Raven P.B. (2003). Carotid baroreflex responsiveness to head-up tilt-induced central hypovolaemia: Effect of aerobic fitness. J. Physiol..

[34] Ogoh S., Nakahara H., Okazaki K., Bailey D.M., Miyamoto T. (2013). Cerebral hypoperfusion modifies the respiratory chemoreflex during orthostatic stress. Clin. Sci..

[35] Jacobi M.S., Patil C.P., Saunders K.B. (1989). Transient, steady-state and rebreathing responses to carbon dioxide in man, at rest and during light exercise. J. Physiol..

[36] Miyamoto T., Bailey D.M., Nakahara H., Ueda S., Inagaki M., Ogoh S. (2014). Manipulation of central blood volume and implications for respiratory control function. Am. J. Physiol. Heart Circ. Physiol..

[37] Poulin M.J., Liang P.J., Robbins P.A. (1998). Fast and slow components of cerebral blood flow response to step decreases in end-tidal PCO_2_ in humans. J. Appl. Physiol..

[38] Mitsis G.D., Poulin M.J., Robbins P.A., Marmarelis V.Z. (2004). Nonlinear modeling of the dynamic effects of arterial pressure and CO_2_ variations on cerebral blood flow in healthy humans. IEEE Trans. Biomed. Eng..

[39] Mitsis G.D., Zhang R., Levine B.D., Marmarelis V.Z. (2006). Cerebral hemodynamics during orthostatic stress assessed by nonlinear modeling. J. Appl. Physiol..

[40] Sobczyk O., Battisti-Charbonney A., Fierstra J., Mandell D.M., Poublanc J., Crawley A.P., Mikulis D.J., Duffin J., Fisher J.A. (2014). A conceptual model for CO(2)-induced redistribution of cerebral blood flow with experimental confirmation using BOLD MRI. Neuroimage.

[41] Lythgoe D.J., Williams S.C., Cullinane M., Markus H.S. (1999). Mapping of cerebrovascular reactivity using BOLD magnetic resonance imaging. Magn. Reson. Imaging.

[42] Toft P.B., Leth H., Lou H.C., Pryds O., Peitersen B., Henriksen O. (1995). Local vascular CO_2_ reactivity in the infant brain assessed by functional MRI. Pediatr. Radiol..

[43] Panerai R.B., Simpson D.M., Deverson S.T., Mahony P., Hayes P., Evans D.H. (2000). Multivariate dynamic analysis of cerebral blood flow regulation in humans. IEEE Trans. Biomed. Eng..

[44] Al-Khazraji B.K., Shoemaker L.N., Gati J.S., Szekeres T., Shoemaker J.K. (2019). Reactivity of larger intracranial arteries using 7 T MRI in young adults. J. Cereb. Blood Flow Metab..

[45] Miller K.B., Howery A.J., Rivera-Rivera L.A., Johnson S.C., Rowley H.A., Wieben O., Barnes J.N. (2019). Age-Related Reductions in Cerebrovascular Reactivity Using 4D Flow MRI. Front. Aging Neurosci..

[46] Giller C.A., Bowman G., Dyer H., Mootz L., Krippner W. (1993). Cerebral arterial diameters during changes in blood pressure and carbon dioxide during craniotomy. Neurosurgery.

[47] Serrador J.M., Picot P.A., Rutt B.K., Shoemaker J.K., Bondar R.L. (2000). MRI measures of middle cerebral artery diameter in conscious humans during simulated orthostasis. Stroke.

[48] Morgan B.J., Crabtree D.C., Palta M., Skatrud J.B. (1995). Combined hypoxia and hypercapnia evokes long-lasting sympathetic activation in humans. J. Appl. Physiol..

[49] Xie A., Skatrud J.B., Crabtree D.C., Puleo D.S., Goodman B.M., Morgan B.J. (2000). Neurocirculatory consequences of intermittent asphyxia in humans. J. Appl. Physiol..

[50] Altose M.D., McCauley W.C., Kelsen S.G., Cherniack N.S. (1977). Effects of hypercapnia and inspiratory flow-resistive loading on respiratory activity in chronic airways obstruction. J. Clin. Investig..

[51] Flenley D.C., Franklin D.H., Millar J.S. (1970). The hypoxic drive to breathing in chronic bronchitis and emphysema. Clin. Sci..

[52] Scano G., Spinelli A., Duranti R., Gorini M., Gigliotti F., Goti P., Milic-Emili J. (1995). Carbon dioxide responsiveness in COPD patients with and without chronic hypercapnia. Eur. Respir. J..

[53] Van de Ven M.J., Colier W.N., Van der Sluijs M.C., Kersten B.T., Oeseburg B., Folgering H. (2001). Ventilatory and cerebrovascular responses in normocapnic and hypercapnic COPD patients. Eur. Respir. J..

[54] Bernardi L., Casucci G., Haider T., Brandstatter E., Pocecco E., Ehrenbourg I., Burtscher M. (2008). Autonomic and cerebrovascular abnormalities in mild COPD are worsened by chronic smoking. Eur. Respir. J..

[55] Hartmann S.E., Pialoux V., Leigh R., Poulin M.J. (2012). Decreased cerebrovascular response to CO_2_ in post-menopausal females with COPD: Role of oxidative stress. Eur. Respir. J..

[56] Lewis N., Gelinas J.C.M., Ainslie P.N., Smirl J.D., Agar G., Melzer B., Rolf J.D., Eves N.D. (2019). Cerebrovascular function in patients with chronic obstructive pulmonary disease: The impact of exercise training. Am. J. Physiol. Heart Circ. Physiol..

